# Modified short axis geometry for left ventricular assessment in patients with hemodynamically significant pulmonary regurgitation

**DOI:** 10.1186/1532-429X-14-S1-T1

**Published:** 2012-02-01

**Authors:** Amy L Tipton, William Gottliebson, Kan N Hor, Joshua Germann, Wojciech Mazur, Woodrow D Benson, Michael Taylor

**Affiliations:** 1 Heart Institute, Cincinnati Children's Hospital Medical Center, Cincinnati, OH, USA; 2 The Heart and Vascular Center, The Christ Hospital, Cincinnati, OH. USA

## Summary

To determine the reliability of MRI LV measurements from a “modified” SA geometry compared with measurements from the standard SA geometry for repaired TOF patients with chronic pulmonary insufficiency.

## Background

Standard MRI assessment of ventricular volume and mass is based on a stack of “standard” short axis images orthogonal to the LV horizontal long axis. Recently, we showed a modified short axis (SA) geometry more accurately defines the tricuspid valve plane, and thereby provides more reliable right ventricular volume estimates in normal subjects and repaired TOF patients. In order to use the modified SA stack as a replacement for the standard SA stack in routine studies, we wanted to prove its reliability for LV measurements.

## Methods

**Figure 1:**
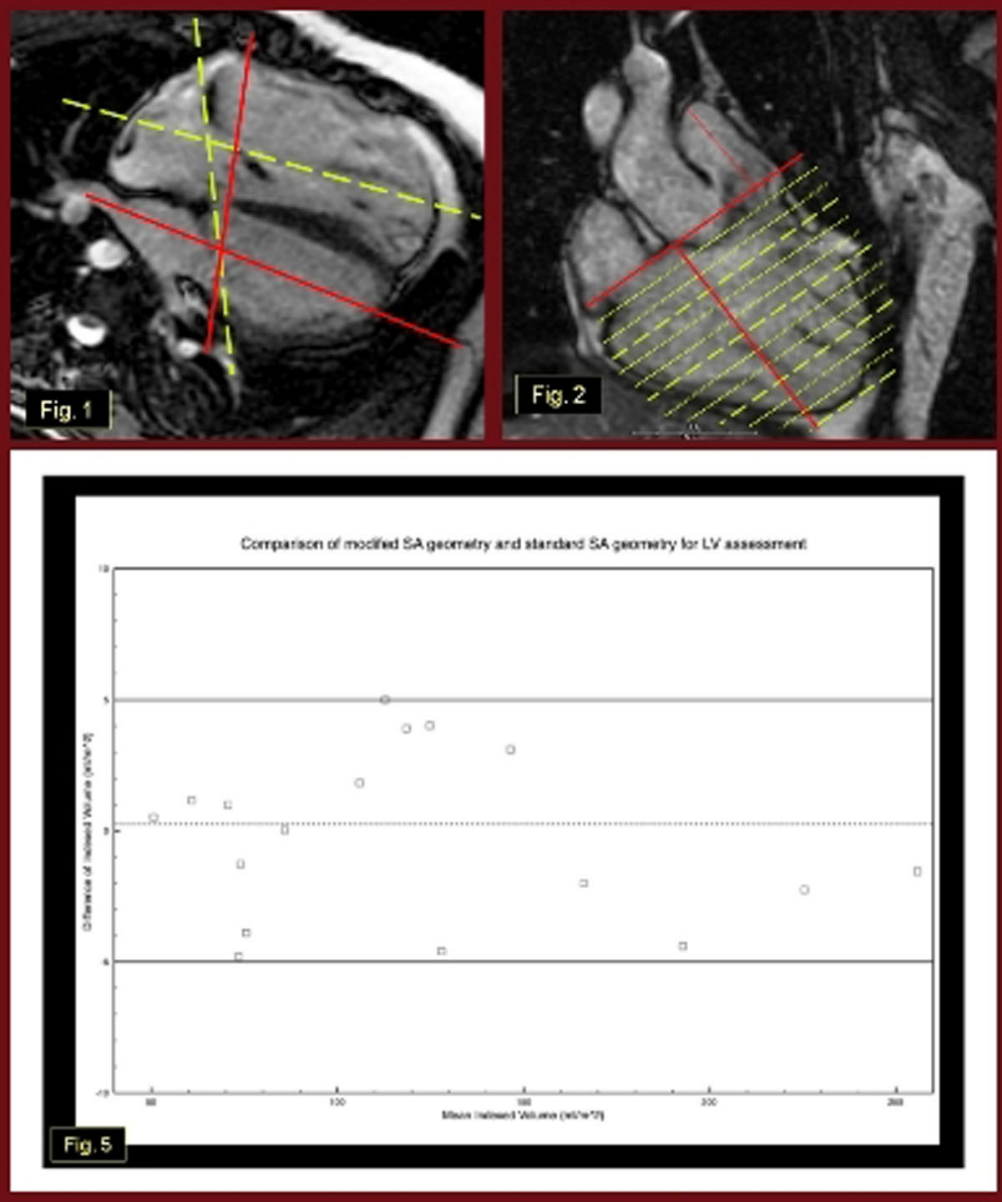


**Figure 2:**
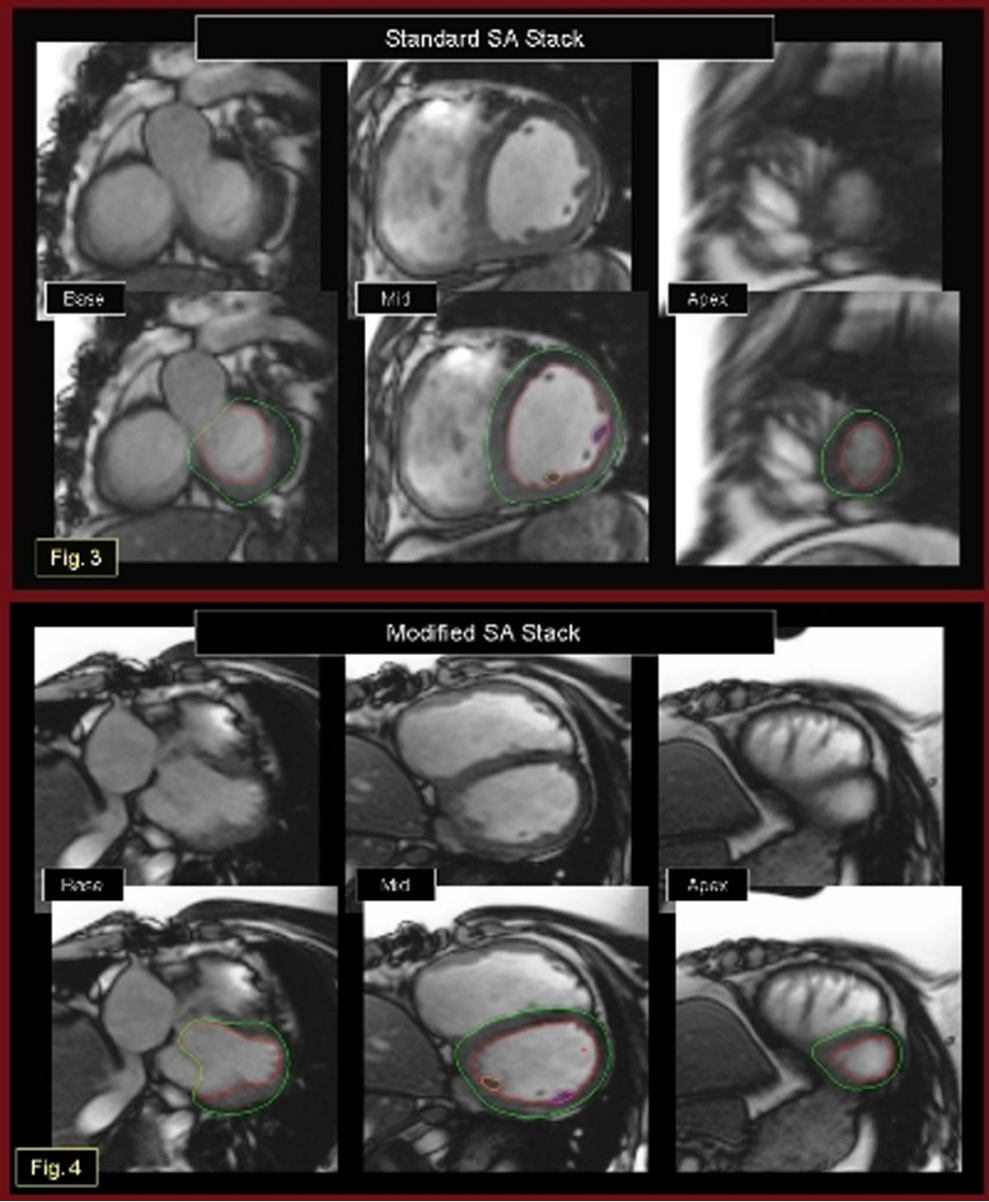


The study was a comparison of cardiac MRI scans of 15 patients (10 male, age range 10 to 25 years) with TOF status post transannular patch repair. Subjects underwent cardiac MRI for standard clinical indications. In addition to standard SA cine SSFP stacks (Fig.1); a second stack of “modified” SA SSFP cines was obtained perpendicular to the RV outflow long axis (Fig.1). LV volumetry and mass were assessed by planimetry of the endocardial and epicardial LV borders at end-systole and end-diastole using MEDIS QMass software. LV volume and mass were measured for both sets of SA stacks (Figs.2-2) by 2 independent expert blinded observers at separate sittings. Agreement between the modified and standard geometry measures was determined by the intraclass correlation coefficients (ICC) and Bland-Altman analysis. Although there are no universal standards, values of ICC of <0.40 are considered low and values of >0.75 are considered high.

## Results

There was excellent agreement for all left ventricular metrics between the standard and the modified SA geometries. The ICC for the two methods was 0.93 (p<0.001) for end-diastolic volume, 0.91 (p<0.001) for end-systolic volume, and 0.91(p<0.001) for LV mass. The Bland-Altman analysis showed no significant bias with limits of agreement <5% (Fig.1(5)). There was also excellent interobserver agreement for the modified short axis geometry with ICCs ranging from 0.91 to 0.95 for the three measures and Bland-Altman limits of agreement <7%.

## Conclusions

The modified SA geometry showed excellent agreement of LV volume measurments compared with the standard SA geometry in this series of chronic RV volume overload patients. These results combined with our previous work showing improved RV assessment by the modified SA geometry makes this the preferred geometry for standard evaluation of repaired TOF patients.

### Abstract Summary Statement

In patients with chronic RV volume overload, a modified short axis geometry that has shown better reliability for RV volumetry also demonstrates excellent agreement for LV volumetry compared with the standard SA geometry.

## Funding

There are no financial disclosures.

